# Long-Term Memory Updating: The Reset-of-Encoding Hypothesis in List-Method Directed Forgetting

**DOI:** 10.3389/fpsyg.2017.02076

**Published:** 2017-11-27

**Authors:** Bernhard Pastötter, Tobias Tempel, Karl-Heinz T. Bäuml

**Affiliations:** ^1^Department of Psychology, University of Trier, Trier, Germany; ^2^Department of Psychology, University of Regensburg, Regensburg, Germany

**Keywords:** long-term memory, episodic memory, directed forgetting, encoding, interference

## Abstract

People’s memory for new information can be enhanced by cuing them to forget older information, as is shown in list-method directed forgetting (LMDF). In this task, people are cued to forget a previously studied list of items (list 1) and to learn a new list of items (list 2) instead. Such cuing typically enhances memory for the list 2 items and reduces memory for the list 1 items, which reflects effective long-term memory updating. This review focuses on the reset-of-encoding (ROE) hypothesis as a theoretical explanation of the list 2 enhancement effect in LMDF. The ROE hypothesis is based on the finding that encoding efficacy typically decreases with number of encoded items and assumes that providing a forget cue after study of some items (e.g., list 1) resets the encoding process and makes encoding of subsequent items (e.g., early list 2 items) as effective as encoding of previously studied (e.g., early list 1) items. The review provides an overview of current evidence for the ROE hypothesis. The evidence arose from recent behavioral, neuroscientific, and modeling studies that examined LMDF on both an item and a list level basis. The findings support the view that ROE plays a critical role for the list 2 enhancement effect in LMDF. Alternative explanations of the effect and the generalizability of ROE to other experimental tasks are discussed.

## Introduction

Long-term memory updating plays a vital role in creating an adaptive human memory system. According to Bjork (1978), such updating is critical because “everyday functioning requires that we keep our (episodic) memories reasonably current. To the degree that we do not somehow set aside or eliminate (irrelevant) information no longer needed we become confused, error prone, and inefficient” (p. 236). Indeed, goal-directed remembering of current or relevant information may fail because irrelevant information is retrieved and thus interferes with the retrieval of the relevant information (Bäuml, 2008). Effective long-term memory updating should reduce interference from irrelevant information and thus promote goal-directed remembering of the current or relevant information.

**FIGURE 1:**
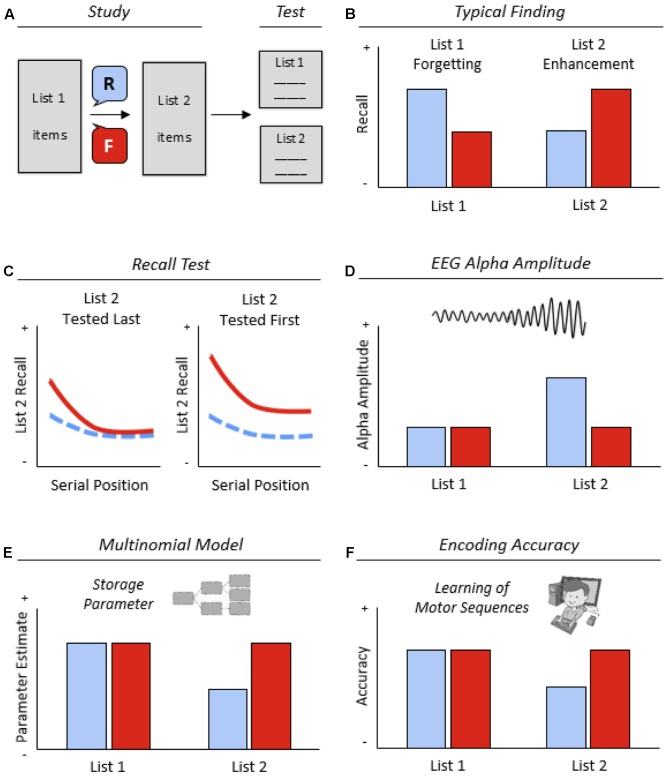
**(A)** The list-method directed forgetting (LMDF) paradigm. Participants study two lists of items and, after the study of list 1 (L1), receive a cue either to forget (F) or continue remembering (R) this list before studying list 2 (L2). After study of L2, a memory test for both lists is conducted, in which participants are asked to recall the items of the two lists, irrespective of original cuing. **(B)** Typical finding. Compared with remember-cued participants, forget-cued participants typically show improved recall of L2 items and impaired recall of L1 items and, referred to as L2 enhancement (L2E) and L1 forgetting (L1F). **(C)** Serial position curves. When L1 is tested first and L2 is tested last, recall enhancement arises for the early L2 items only; when L2 is tested first, recall enhancement arises for all L2 items, although the early L2 items show a larger enhancement effect than the middle and late L2 items. F, solid line; R, dashed line. **(D)** Neurocognitive evidence for reset-of-encoding (ROE). EEG alpha amplitude during item encoding increases from L1 to L2 in the remember condition, but does not change in the forget condition. Low alpha amplitude indicates high encoding efficiency. **(E)** Model-based evidence for ROE. The estimate of the encoding parameter decreases from L1 to L2 in the remember condition, but does not change in the forget condition. **(F)** Behavioral evidence for ROE. In motor sequence learning, encoding accuracy decreases from L1 to L2 in the remember condition, but does not change in the forget condition.

In the laboratory, memory updating can be studied with the list-method directed forgetting (LMDF) paradigm (Bjork, 1970, 1972). In this paradigm, participants study two lists of items (e.g., words, sentences, or pictures) and, after study of list 1 (L1), receive a cue either to forget or to continue remembering this list (**Figure 1A**). After study of list 2 (L2) and a short retention interval, a memory test for both lists is conducted, in which all participants are asked to recall the items of the two lists, irrespective of original cuing. The typical finding is that forget-cued participants recall more L2 items and fewer L1 items than remember-cued participants. The two effects of the forget cue are referred to as *L2 enhancement* (L2E) and *L1 forgetting* (L1F) in the following (**Figure 1B**; MacLeod, 1998; Bäuml et al., 2010; Sahakyan et al., 2013). This review focuses on the cognitive mechanisms underlying L2E, that is, the beneficial effect of intentional memory updating in the LMDF paradigm.

## From Single- To Dual-Mechanism Accounts of LMDF

Single-mechanism accounts of LMDF assume that L2E and L1F are the two sides of the same coin and are mediated by the same cognitive mechanism. For instance, the retrieval-inhibition account assumes that participants engage in active inhibitory processes in response to the forget cue. The inhibition impairs access to L1 context and, due to the resulting decrease in L1 items’ interference level, enhances memory for L2 (Geiselman et al., 1983). Alternatively, the context-change account assumes that forget-cued participants deliberately change mental context between study of the two lists. Such change may impair access to L1 context, reduce the list’s interference level, and thus improve memory for L2 (Sahakyan and Kelley, 2002). Although the two single-mechanism accounts thus differ in detail with regard to the exact nature of the mediating mechanism, they both take a retrieval view on L2E, attributing the effect to interference reduction at test (for further single-mechanism accounts, see MacLeod, 1998).

Several findings are in line with single-mechanism accounts of LMDF and the retrieval view on L2E. Bäuml and Kliegl (2013), for instance, showed that the forget cue reduces response latency during L2 recall and makes it similar to latency when a single list has been studied only. Because reduced response latency is assumed to reflect a reduction in participants’ memory search set size (Wixted and Rohrer, 1993), the finding indicates more focused memory search during L2 recall in forget-cued participants, as caused by reduced interference from L1 (see also Bjork and Bjork, 1996; Sahakyan and Kelley, 2002). In contrast, there are also findings that are inconsistent with single-mechanism accounts of LMDF, for instance, reporting dissociations between L2E and L1F. These dissociations indicate that L2E can occur without L1F (e.g., Pastötter et al., 2016; Tempel and Frings, 2016) and L1F can occur without L2E (e.g., Sahakyan and Delaney, 2003; Pastötter and Bäuml, 2010). To explain these findings, dual-mechanism accounts of LMDF have been suggested, according to which both retrieval and encoding processes can contribute to LMDF (Sahakyan and Delaney, 2003; Pastötter and Bäuml, 2010; Pastötter et al., 2012).

One hypothesis reflecting such view is reset-of-encoding (ROE; Pastötter and Bäuml, 2010; Pastötter et al., 2012). The ROE hypothesis is based on the view that encoding efficacy of studied items decreases with number of encoded items, and is thus better for early than for later studied items. Such decrease may arise due to increases in working memory load or reduced attention during encoding when more and more items are encoded (Sederberg et al., 2006; Pastötter et al., 2008). Within a study list, this view is reflected in the well-known primacy effect, which demonstrates better memory for early than for middle and late studied list items (Deese and Kaufman, 1957; Murdock, 1962). The view is also reflected in the generally better memory for a first compared to a second study list, when two item lists were encoded in succession. Here the effect is mainly caused by the presence of a primacy effect in the first list and the absence (or reduction) of the effect in the second (e.g., Geiselman et al., 1983; Pastötter and Bäuml, 2010). The critical assumption of the ROE hypothesis then is that providing a forget cue after study of L1 resets the encoding process and makes the encoding of the early L2 items as effective as the encoding of the early L1 items. Two immediate predictions arise from such a view, one on an item and the other on a list level basis: The one prediction is that, after a forget cue, not only L1 but also L2 should show a primacy effect, leading to better memory for early than later studied L2 items. The other prediction is that (neural and modeling) parameters relating to encoding efficiency should change from L1 to L2 encoding in the remember condition but not in the forget condition.

The ROE hypothesis has been incorporated in the dual-mechanism account of LMDF of Pastötter et al. (2012). According to this account, two mechanisms contribute to LMDF: a retrieval mechanism, i.e., retrieval inhibition, supposed to contribute to both L2E and L1F; and an encoding mechanism, i.e., ROE, supposed to contribute to L2E (of early L2 items) only. In the next paragraphs, we will focus on L2E and review current evidence for the ROE hypothesis.

## Evidence for ROE

First evidence for the ROE hypothesis arose from behavioral studies that examined items’ serial position curves in LMDF on an item level basis. In more recent work, additional evidence for the ROE hypothesis emerged from neurocognitive, model-based, and motor learning studies addressing LMDF on a list level basis. We will review both lines of evidence for ROE.

### Evidence from Serial Position Curves

First evidence that the forget cue in LMDF may have a selective enhancement effect for early L2 items arose in the studies by Geiselman et al. (1983) and Sahakyan and Foster (2009), reporting improved recall of early relative to later L2 items. On the basis of these findings, Pastötter and Bäuml (2010) ran a series of new LMDF experiments, in which they manipulated number and presentation rate of L2 items. At test, participants were instructed to recall L1 items first and L2 items second. Analysis of items’ serial position curves showed a large recall enhancement effect for the early L2 items, but no reliable enhancement effect for the middle and late L2 items (**Figure 1C**). Neither number nor presentation rate of L2 items influenced the selective enhancement effect for the early L2 items. On the basis of these findings, the ROE hypothesis was suggested, attributing the selective enhancement effect to a reset of the encoding process in response to the forget cue.^1^

Further evidence for the ROE hypothesis arose from a study that examined the influence of the two lists’ recall order at test for LMDF effects (Pastötter et al., 2012). When L1 was recalled first and L2 was recalled second, a selective enhancement effect for the early L2 items arose, replicating previous findings by Pastötter and Bäuml (2010). In contrast, when L2 was recalled first, recall enhancement for all L2 items arose, although the early L2 items stilled showed a larger enhancement effect than did the middle and late L2 items (**Figure 1C**). These findings indicate that two factors can contribute to L2E: one factor that pertains to early L2 items only and is present regardless of list recall order, and a second factor that pertains to all L2 items and is present only if L2 is recalled first. The dual-mechanism account of Pastötter et al. (2012) suggests that the first factor is ROE and the second factor is interference reduction due to retrieval inhibition. In fact, interference reduction for (all) L2 items may be reduced when L1 is recalled first, because the preceding recall of L1 items can reactivate L1 study context and thus reinstate L1 items’ interference potential (Bäuml and Samenieh, 2010, 2012).

Pastötter et al. (2016) reported further support for the ROE hypothesis by examining serial position effects when employing item recognition tests. In this study, a large enhancement effect in item recognition for the early L2 items was found, but no enhancement effects for the middle and late L2 items emerged, irrespective of list testing order. Because item recognition should be sensitive to ROE-induced improved encoding but be fairly insensitive to (a reduction in) proactive interference (see Kinnell and Dennis, 2011), the finding by Pastötter et al. (2016) corroborates the view that the selective enhancement effect for the early L2 items reflects ROE and is in line with Pastötter et al.’s (2012) dual-mechanism account.

### Neurocognitive Evidence

Neurocognitive evidence for the ROE hypothesis arose from LMDF studies that examined EEG alpha oscillations during the encoding of the two item lists (Bäuml et al., 2008; Hanslmayr et al., 2012). In prior EEG work, brain oscillations at distinct frequencies were linked to memory function (Nyhus and Curran, 2010; Hanslmayr et al., 2016). In particular, in episodic memory, EEG alpha oscillations (8–14 Hz) were associated with encoding efficiency in both single- and multi-list studies, with increases of EEG alpha amplitude indicating impaired item encoding (Sederberg et al., 2006). Consistently, in several studies, alpha amplitude was found to increase with number of encoded items, which was attributed to increases in memory load and reduced attention during item encoding (Sederberg et al., 2006; Pastötter et al., 2008, 2011).

Examining the role of EEG alpha oscillations in LMDF, Hanslmayr et al. (2012) demonstrated that alpha amplitude during item encoding increases from L1 to L2 in the remember condition, but not in the forget condition (**Figure 1D**). In addition, Bäuml et al. (2008) showed that the difference in EEG alpha amplitude during L2 encoding between the forget and remember conditions is specifically related to L2E, but not to L1F. Together, these neurocognitive findings indicate that the forget cue resets neural activity back to L1 level and thus improves the encoding (and remembering) of L2 items, which fits with the ROE hypothesis.

### Model-Based Evidence

Model-based evidence for the ROE hypothesis arose from a study using multinomial modeling to investigate LMDF (Rummel et al., 2016). Multinomial models are a class of mathematical models that can be used to disentangle the cognitive processes underlying observable behavioral effects on the basis of categorical data (Batchelder and Riefer, 1999). Rummel et al. (2016) applied the storage-retrieval model (Rouder and Batchelder, 1998) to quantify the relative contribution of encoding and retrieval processes to LMDF. Two modifications of the standard paradigm were necessary to meet the model’s assumptions. First, participants studied word pairs instead of single words and, second, the usual free recall test was followed by an additional cued recall test. In the free recall test, L1 was tested first and L2 was tested second.

Recall results demonstrated both reliable L2E and L1F in the free recall test. In the model-based analysis, both storage and retrieval parameters were estimated. Estimates of the storage parameter were specifically related to L2E, whereas estimates of the retrieval parameter were specifically related to L1F. The storage parameter decreased from L1 to L2 in the remember condition, but did not change in the forget condition (**Figure 1E**). The finding suggests impaired encoding from L1 to L2 in the remember condition but not in the forget condition, which is consistent with the ROE hypothesis. Moreover, the model-based dissociation between L2E and L1F supports dual-mechanism views on LMDF that attribute LMDF effects to different mechanisms.

### Evidence from Motor Sequence Learning

Further direct evidence for the ROE hypothesis comes from a LMDF study by Tempel and Frings (2016) that investigated the effects of cuing on motor sequence learning on a list level basis. Participants learned two lists of sequential finger movements (SFMs). Each list consisted of five SFMs and each SFM consisted of four key presses on a computer keyboard, performed with three fingers of the right hand. Both accuracy in entering SFMs in the learning phase and correct recall of SFMs in the test phase were analyzed as a function of cuing condition, separately for the two lists. L2 was tested after L1. Three results emerged. First, encoding accuracy decreased from L1 to L2 in the remember condition, but did not change in the forget condition (**Figure 1F**). Second, forget-cued participants correctly recalled more L2 SFMs than remember-cued participants. Third, L2 encoding accuracy mediated L2 recall enhancement. The findings indicate that LMDF effects generalize from verbal learning to non-verbal motor learning. In particular, they support the ROE hypothesis, suggesting that the forget cue resets motor encoding quality for L2 SFMs back to L1 level.

## Alternative Encoding Hypotheses

In addition to the ROE hypothesis, two other hypotheses exist in the LMDF literature that attribute L2E to beneficial encoding: the strategy-change hypothesis and the selective-rehearsal hypothesis. The strategy-change hypothesis assumes that L2E arises from a change in forget-cued participants’ encoding strategy (Sahakyan and Delaney, 2003). The idea is that the forget cue induces evaluation of participants’ L1 encoding strategy and a shift to a better L2 encoding strategy, leading to more elaborate encoding of L2 items in the forget than in the remember condition (Sahakyan et al., 2004). Because, arguably, different encoding strategies should affect the encoding for all L2 items (Glanzer and Koppenaal, 1977), the strategy-change hypothesis predicts non-selective enhancement for all L2 items, which is inconsistent with the finding of a selective enhancement effect for the early L2 items. To reconcile the hypothesis with this finding, the restriction would have to be made that forget-cued participants shift to an encoding strategy that is beneficial for early L2 items only, or shift to more elaborate encoding for early L2 items and then switch back to less elaborate encoding for middle and late L2 items, which of course contrasts with Glanzer and Koppenaal’s (1977) finding.

The selective-rehearsal hypothesis assumes that during L2 encoding remember-cued participants rehearse both L1 and L2 items in working memory, whereas forget-cued participants rehearse L2 items only, which improves subsequent memory for L2 at the expense of L1 (Bjork, 1970). On the item level, the hypothesis assumes that the enhancement should be largest for early L2 items and the forgetting should be largest for late L1 items, claiming that, once the forget cue is provided, mainly the late L1 items are deleted from the rehearsal buffer and the rehearsal starts over again with the encoding of the early L2 items (Bruce and Papay, 1970). Although the selective-rehearsal hypothesis is consistent with the finding of a selective enhancement effect for the early L2 items, it is inconsistent with the findings that (i) L1F is typically absent in item recognition (e.g., Benjamin, 2006; Pastötter et al., 2016), and (ii) L1F is typically non-selective in recall tests (e.g., Sahakyan and Foster, 2009; Pastötter and Bäuml, 2010).

## From LMDF to Other Experimental Tasks

The ROE hypothesis may not be restricted to the LMDF paradigm but generalize to other experimental tasks. For instance, several findings suggest that ROE can play a role in experimental tasks involving retrieval practice. Retrieval practice can have a number of beneficial effects for memory and learning (see Roediger et al., 2011). One such effect, referred to as the forward effect of testing in the literature, is that retrieval practice of previously studied information can increase retention of subsequently studied new information (Szpunar et al., 2008; for a review, see Pastötter and Bäuml, 2014). Arguably, the forward effect may partly be mediated by some form of self-induced forget instruction, inducing participants to think that the practiced information is no longer needed and thus can be forgotten (Szpunar et al., 2007; see also Abel and Bäuml, 2016). If so, retrieval practice should show the same basic effects as a forget cue, including ROE. Results from EEG studies support the proposal showing that retrieval practice (of episodic, semantic, and autobiographical memories) between the study of lists can disrupt alpha amplitude increases from the encoding of earlier to later lists, which may reflect ROE on a list level basis (Pastötter et al., 2008, 2011). To examine the role of ROE for the forward effect of testing or other experimental tasks more thoroughly, future work is needed to investigate the presence of ROE on an item level basis.

## Conclusion

Several lines of LMDF studies support the ROE hypothesis. First, on the list level, there is consistent evidence from neurocognitive, model-based, and motor-learning studies indicating that the forget cue can reset post-cue encoding processes, making the encoding of L2 items about as effective as the encoding of L1 items. Second, on the item level, there is conclusive evidence from analysis of serial position data showing that, in response to a forget cue, L2 items at early serial learning positions are subject to selective enhancement and thus show a primacy effect similar to early L1 items. All of these findings converge on the view that ROE plays a critical role in LMDF and long-term memory updating. Finally, there is evidence that ROE can also be involved in other experimental tasks, which points to a more general role of ROE in creating an adaptive memory system.

## Author Contributions

BP drafted the manuscript. TT and K-HB provided critical revisions.

## Conflict of Interest Statement

The authors declare that the research was conducted in the absence of any commercial or financial relationships that could be construed as a potential conflict of interest.

## Footnotes

1 Note that, regarding L1, no influence of items’ serial learning position was observed. Indeed, all L1 items showed about the same amount of forgetting, which is consistent with the idea that L1F arises from reduced L1 context access (e.g., Sahakyan and Foster, 2009; Pastötter and Bäuml, 2010).
